# Draft Genome Sequence of a Primate Isolate of *Kazachstania pintolopesii*

**DOI:** 10.1007/s11046-023-00772-8

**Published:** 2023-08-17

**Authors:** Steve A. James, Aimee Parker, Catherine Purse, Andrea Telatin, David Baker, Rhiannon Evans, Sandy Holmes, Simon G. P. Funnell, Simon R. Carding

**Affiliations:** 1 Food, Microbiome and Health, Quadram Institute Bioscience, Norwich, UK; 2 UK Health Security Agency, Porton Down, Salisbury, UK; 3 Norwich Medical School, University of East Anglia, Norwich, UK

**Keywords:** *Kazachstania pintolopesii*, Draft genome, Non-human primate, Cynomolgus macaque, Fungal pathobiont, Gut mycobiota

## Abstract

*Kazachstania pintolopesii* is an opportunistic mammalian pathobiont from the *K. telluris* species complex. No draft genomes of this species are currently available. Here, we report the first draft genome sequence of a primate isolate of *K. pintolopesii* (NCYC 4417).

*Kazachstania pintolopesii* is an ascomycetous yeast and relative of *K. bovina*, *K. heterogenica*, *K. slooffiae* and *K. telluris* [1]. Collectively, these species constitute the *K. telluris* complex, a phylogenetically distinct group of thermotolerant yeasts able to grow at 37 °C and above [1, 2]. They are widely distributed [1], but are found predominantly in the gastrointestinal (GI) tracts and nasal passages of birds and mammals [1–3]. Rodents are the principal hosts for *K. pintolopesii* [1, 2, 4] with a recent study extending its host range to include cynomolgus macaques [5]. These fungi are also considered pathobionts causing infections in rodents, primates, and less frequently in humans [2, 6, 7]. *K. pintolopesii* is in addition to being a gut commensal also associated with fatal infections in laboratory mice [2], and with ankylosing spondylitis in cynomolgus macaques [8]. Draft genomes for three members of the complex have been published [9–11], but despite its potential significance as a pathobiont no *K. pintolopesii* genomes have been published to date.

Here, we have combined short- and long-read sequencing to obtain the genome sequence of *K. pintolopesii* NCYC 4417, a faeces-derived isolate from a captive adult macaque. A faecal homogenate was prepared in sterile phosphate-buffered saline (PBS) and cultured onto Sabouraud dextrose (SD) agar plates containing penicillin (25 U/mL) and streptomycin (25 U/mL) at 37 °C. Species identity, from single colonies, was determined by PCR amplification and Sanger sequencing of the ribosomal DNA internal transcribed spacer 1 (ITS1) region of the ribosomal DNA locus using primers ITS1F [12] and ITS2 [13]. The ITS1 sequence of strain NCYC 4417 (GenBank accession number PRJEB63679) is 99.7% identical to that of the *K. pintolopesii* type strain CBS 2985 (GenBank accession number NR_155233).

For short- and long-read sequencing, total genomic DNA was extracted from a stationary phase SD culture using a MasterPure Yeast DNA Purification Kit (Cambio, Cambridge, UK). Short-read Illumina sequencing was performed using a modified 20-fold dilution of DNA Prep (Flex) reagent and run on a NextSeq 500 sequencer, producing 9,108,736 paired-end 150-bp reads (~ 186 × coverage). Nanopore sequencing was performed using a MinION sequencer (Oxford Nanopore Technologies, ONT), ligation sequencing kit SQK-LSK109 (ONT) and flow cell FLO-MIN106 R9.4.1 (ONT). This produced a total of 437,446 reads with an average read length of 4,069 bases (~ 127 × coverage). Base calling was performed using Guppy v.6.1.2 (basecall_model_version_id = 2021–05-17_dna_r9.4.1_minion_384_d37a2ab9). Raw short- and long-read polishing, including the removal of adapters and low-quality bases, was performed using SeqFu 1.16 [14] and fastp 0.23 [15]. The genome was assembled using Flye 2.9.1 [16] and polished with one round of Pilon 1.24 [17]. The genome assembly comprised of 34 contigs with the largest being 1,738,127 bp in length. The total size of the genome was 13,992,981 bp, the *N*_50_ value was 947 kb, and the G + C content was 30.63%. In addition, a putative 8-nt telomeric repeat was identified (5’-WGTATGGG-3’), similar in sequence to the canonical eukaryotic telomere motif [18], and present in 50–100 tandem copies at one or both termini of 13 contigs. Augustus v.3.3.3 [19] predicted 4884 protein-coding genes using the *Saccharomyces cerevisiae* (S288C) training data set, and 196 tRNA genes were detected using tRNAscan-SE 2.0 [20]. Genome completeness was estimated as 91.0% using BUSCO v5.4.4 [21]. Dependencies and scripts are available at https://github.com/quadram-institute-bioscience/ont-candida.

## Data Availability

This whole-genome shotgun project has been deposited at DDJB/ENA/Genbank (Biosample Number SAMEA112953918, BioProject Number PRJEB61520, and Assembly Accession Number GCA_950065675.1). The version described in this paper is version 1. The raw reads were deposited at SRA (Accession Numbers ERR11267923 and ERR11267961). This article was written according to MycopathologiaGENOMES checklist requirements [22].
